# Trends in Synchronous Teledermatology Use by Level of Service Among Medicare Beneficiaries From 2017 to 2023: Retrospective Observational Study

**DOI:** 10.2196/78438

**Published:** 2025-12-15

**Authors:** Vincent Le, Carina Shiau, Arash Pour Mohammad, Vivian Yang, Gordon Hyeonjin Bae

**Affiliations:** 1 School of Medicine, Georgetown University, Washington, DC, United States; 2 Harvard Medical College, Harvard University, Boston, MA, United States; 3 Department of Dermatology, School of Medicine, Stanford University, 450 Broadway Street, Pavilion C, 2nd Floor, Redwood City, CA, 94063, United States, 1 (650) 721-7190; 4 School of Medicine, University of California, San Francisco, San Francisco, CA, United States

**Keywords:** teledermatology, evaluation and management, Medicare, telemedicine, telehealth, dermatology, visit complexity, level of service

## Abstract

This retrospective observational study was conducted to evaluate trends in synchronous teledermatology use across different levels of clinical complexity among Medicare beneficiaries, analyzing data from the Medicare Part B Physician/Supplier Procedure Summary Master Files; we founding that the growing use of teledermatology for complex visits highlights its potential as a long-term approach for improving dermatologic care.

## Introduction

The use of teledermatology has significantly increased since the onset of the COVID-19 pandemic. Teledermatology provides several benefits, including improving access to care for underserved communities and saving patients time when in-person visits are not medically necessary [1]. This modality has been integrated into the care of medically complex patients (levels of service 4 and 5) and serves as a valuable tool for managing their conditions [2]. However, there remains a limited understanding of teledermatology use across varying levels of clinical complexity. Levels of service refer to medical billing categories that describe the complexity involved in a patient visit, ranging from 1 (brief) through 5 (long, with greater clinical judgment). In this study, we explore trends in synchronous teledermatology use across levels of service, providing insights that can inform the clinical integration of teledermatology.

## Methods

### Study Design

We conducted a retrospective observational study using the Medicare Part B Physician/Supplier Procedure Summary Master Files from 2017 to 2023 [3]. Outpatient evaluation and management, in-person visits, and teledermatology visits billed by dermatologists were identified via Current Procedural Terminology codes and stratified by service level (levels 1‐5) and patient type (new vs established). Percentages of visits by service level were calculated to characterize trends before and after the pandemic. Descriptions of the data collection methodology and statistical analysis are provided in Multimedia Appendix 1).

### Ethical Considerations

This was a secondary analysis of existing data that did not involve interaction with human subjects and used deidentified data. This retrospective study is thus exempt from informed consent and institutional review board approval.

## Results

**Figure 1.**
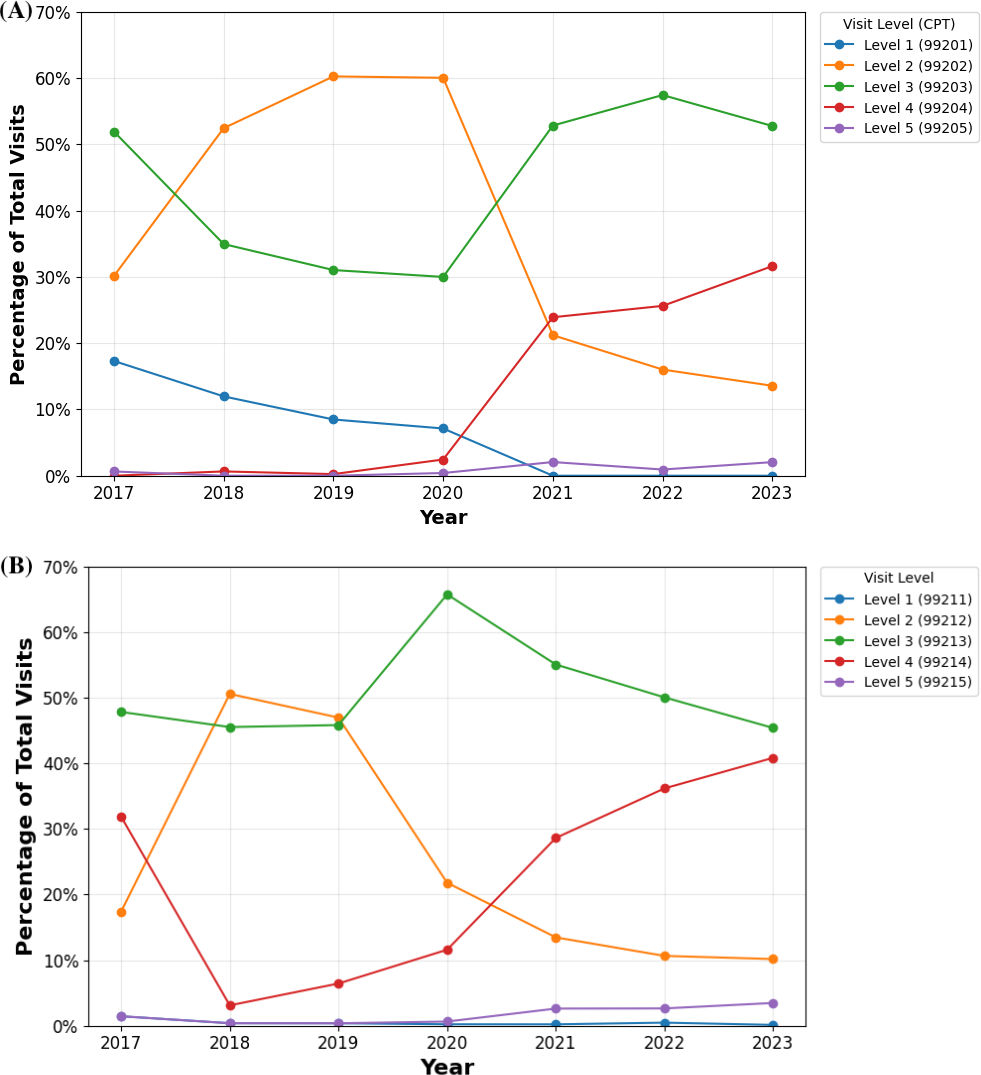
(**A**) Percentage of new-patient teledermatology visits by level of service from 2017 to 2023 among Medicare beneficiaries. (**B**) Percentage of established-patient teledermatology visits stratified by level of service from 2017 to 2023 among Medicare beneficiaries. CPT: Current Procedural Terminology.

**Figure 2.**
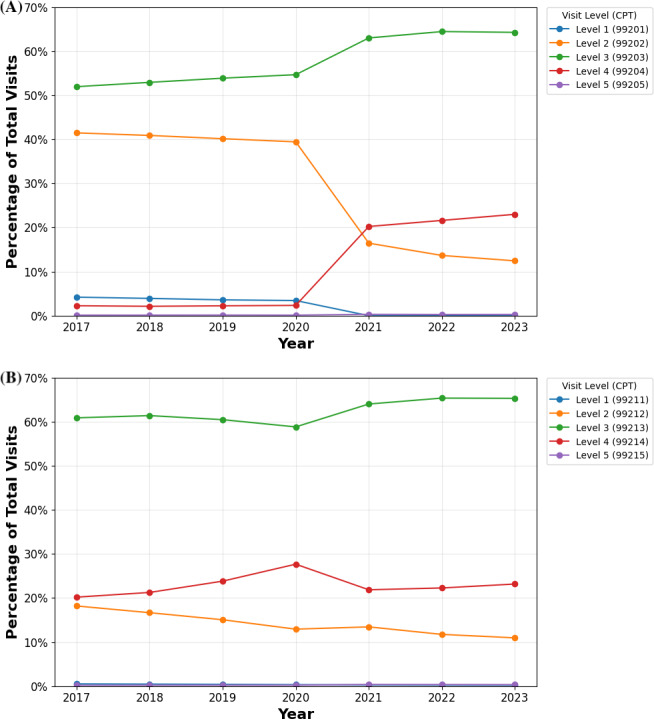
(**A**) Percentage of new-patient in-person visits stratified by level of service from 2017 to 2023 among Medicare beneficiaries. (**B**) Percentage of established-patient in-person visits stratified by level of service from 2017 to 2023 among Medicare beneficiaries. CPT: Current Procedural Terminology.

Before 2020, level 2 and 3 visits accounted for the highest proportion of teledermatology visits among both patient groups (Figure 1). From 2020 to 2023, there was an increase in teledermatology use for level 4 and 5 visits among new patients at level 4 (2020: 570/22,571, 2.53%; 2021: 1468/6069, 24.19%; 2022: 838/3170, 26.44%; and 2023: 814/2468, 32.98%) and level 5 (2020: 115/22,571, 0.51%; 2021: 137/6069, 2.26%; 2022: 32/3170, 1.01%; and 2023: 52/2468, 2.11%), as well as for established patients at level 4 (2020: 21,594/184,044, 11.73%; 2021: 14,337/49,666, 28.87%; 2022: 10,417/28,643, 36.37%; and 2023: 9524/23,329, 40.82%) and level 5 (2020: 1352/184,044, 0.73%; 2021: 1422/49,666, 2.86%; 2022: 826/28,643, 2.88%; and 2023: 867/23,329, 3.72%). In contrast, level 2 teledermatology visits decreased from 2020 to 2023 for new (2020: 13,506/22,571, 59.84%; 2021: 1280/6069, 21.10%; 2022: 503/3170, 15.87%; and 2023: 330/2468, 13.37%) and established (2020: 39,881/184,044, 21.67%; 2021: 6632/49,666, 13.35%; 2022: 3009/28,643, 10.51%; and 2023: 2371/23,329, 10.16%) patients. Compared to teledermatology visits from the same period, in-person visits had a lower percentage of complex visits (levels 4 and 5) for both patient types (Figure 2). Although in-person level 4 and 5 new-patient visits increased, their growth remained lower than for teledermatology. Among established patients, in-person visits showed a level 4 decline and slight level 5 increase, contrasting the growth in teledermatology for these levels. Due to these trends, in 2023, the level 4 and 5 visits among total teledermatology visits numbered 814 of 2468 (32.3%) and 52 of 2468 (2.11%) among new patients and 9524 of 23,329 (40.82%) and 867 of 23,329(3.82%) among established patients, respectively. In comparison, for in-person visits, level 4 and 5 visits accounted for 308,577 of 1,342,022 (22.99%) and 3727 of 1,342,022 (0.28%) of visits among new patients, and 2,130,561 of 9,196,175 (23.17%) and 31,858 of 9,196,175 (0.35%) among established patients (Multimedia Appendix 2).

## Discussion

Our study identified that early on, teledermatology focused on lower-level visits (levels 2 and 3), but shifted toward higher-level encounters (levels 4 and 5) after 2020. This trend toward higher-level visits may be driven by multiple factors. One possible factor is the 2021 evaluation and management billing change, enabling health care providers to determine visit level by time or by medical decision [4]. This change may have simplified justification for higher-level virtual visits, leading to coding changes. However, the greater growth of higher-level encounters in teledermatology compared to in-person visits suggests factors beyond billing requirements contributed to this trend. The COVID-19 pandemic led to greater reliance on teledermatology, likely increasing dermatologist comfort with using this modality for managing complex conditions that do not require in-person visits [5]. However, as total teledermatology visits declined from 2020 to 2023, dermatologists still using virtual visits were likely those with greater comfort using telehealth or medical systems that easily incorporate virtual care. Our findings highlight important considerations for policymakers and health systems. For policymakers, coding trends can inform future telehealth billing changes to balance access, quality, and cost. For health systems, the shift highlights the importance of integrating teledermatology into practice for effective use.

Our study is limited in its capacity to explore the multiple factors that influenced the findings and by its sole examination of Medicare beneficiaries, which may not have captured the entirety of teledermatology use, reducing generalizability. Limitations in the dataset may have led to undercounting teledermatology visits, as only the first and second modifier fields were available. Furthermore, teledermatology is often used in settings where health care providers feel comfortable with virtual care, potentially skewing the patient population. Further research is needed to continue evaluating the scope of teledermatology in dermatologic care and improve our understanding of the factors that influence its increasing use for complex visits.

## Supplementary material

10.2196/78438 Multimedia Appendix 1 Data collection methodology and statistical analysis.

10.2196/78438 Multimedia Appendix 2 Total evaluatation and management and teledermatology visit count data tables.

## References

[1] Maddukuri S, Patel J, Lipoff JB (2021). Teledermatology addressing disparities in health care access: a review. Curr Dermatol Rep.

[2] Kazi R, Evankovich MR, Liu R (2021). Utilization of asynchronous and synchronous teledermatology in a large health care system during the COVID-19 pandemic. Telemed J E Health.

[3] Physician/supplier procedure summary. Centers for Medicare & Medicaid Services.

[4] (2021). CPT evaluation and management (E/M) office or other outpatient (99202-99215) and prolonged services (99354, 99355, 99356, 99417) code and guideline changes. American Medical Association.

[5] Gronbeck C, Grant-Kels JM, Lu J, Feng H (2022). Increased utilization of teledermatology among Medicare Part B beneficiaries during the COVID-19 pandemic. Clin Dermatol.

