# Supplementary material for: Trends in Synchronous Teledermatology Use by Level of Service Among Medicare Beneficiaries From 2017 to 2023: Retrospective Observational Study
Source: JMIR Dermatol. 2025 Dec 15;8:e78438. doi: 10.2196/78438 (PMC12704911; doi:10.2196/78438)
Supplement: Multimedia Appendix 1 [file derma-v8-e78438-s001.docx]

### **Methods**

*Patient cohort and data sources*

We conducted a retrospective study using electronic health record data from the Centers for

Medicare and Medicaid Services (CMS) Chronic Conditions Data Warehouse. The dataset

captures CMS administrative claims information on services and procedures provided to Original

Medicare Part B beneficiaries by physicians and healthcare professionals nationwide from 2017

to 2023.

We identified dermatologists using the provider specialty code equal to “07” on the part B claims. To filter for all evaluation and management (E&M) office visits among dermatologists, we identified all claims billed for the healthcare common procedure coding system (HCPCS) codes “99201-99205” and “99211-99215” by dermatologists. We further identified teledermatology visits by identifying all E&M visits billed with an initial modifier equal to “95” or place of service code equal to “2” or “10.” These modifiers within the claims allowed us to differentiate between an in-person and virtual visit. We further distinguished new patient visits from established patient visits, which were divided by HCPCS codes “99201-99205” and “99211-99215” respectively. Finally, we stratified teledermatology visits by levels of service, with “99201-99205” corresponding to increasing levels of complexity for new patients visits and “99211-99215” for established patients visits. The difference between total E&M visits and teledermatology visits for each year was calculated to identify in-person visit counts and further separated to patient type and level of visits. Total counts were calculated using the sum of the physician submitted service counts to get the total number of teledermatology visits.

***Statistical analysis***


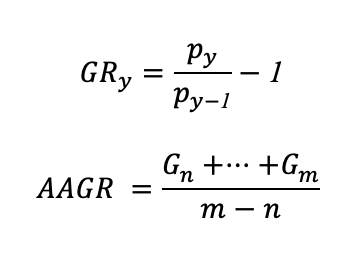
We evaluate the total number and proportion of in-person and teledermatology services provided by dermatologists from 2017 to 2023. To compute the average annual growth rate (AAGR) for the time frames 2017-2019 and 2020-2023 for each level of service, we use the following formula:

$${GR}_{y}=\frac{p_{y}}{p_{y-1}}-1$$

$$AAGR =\frac{G_{n}+\cdot\cdot\cdot+G_{m}}{m-n}$$

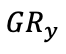
where the growth rate ${GR}_{y}$ between years *y* and *y-1* is determined by the ratio between the proportion *p* of teledermatology visits conducted by dermatologists between each respective year. All analyses were conducted using Python, and a p-value < 0.05 was considered statistically significant.
